# Factors associated with the composition and diversity of the cervical microbiota of reproductive-age Black South African women: a retrospective cross-sectional study

**DOI:** 10.7717/peerj.7488

**Published:** 2019-08-15

**Authors:** Harris Onywera, Anna-Lise Williamson, Zizipho Z.A. Mbulawa, David Coetzee, Tracy L. Meiring

**Affiliations:** 1 Institute of Infectious Disease and Molecular Medicine, University of Cape Town, Cape Town, Western Cape, South Africa; 2 Division of Medical Virology, Department of Pathology, Faculty of Health Sciences, University of Cape Town, Cape Town, Western Cape, South Africa; 3 SAMRC Gynaecological Cancer Research Centre, University of Cape Town, Cape Town, Western Cape, South Africa; 4 Center for HIV & STIs, National Institute for Communicable Diseases, National Health Laboratory Service, University of Cape Town, Johannesburg, Gauteng, South Africa; 5 Center for Infectious Disease Epidemiology and Research, School of Public Health and Family Medicine, University of Cape Town, Cape Town, Western Cape, South Africa

**Keywords:** Cervical microbiota, Bacterial vaginosis (BV), Human papillomavirus (HPV), Hormonal contraception, Black South African, Reproductive-age

## Abstract

**Background:**

*Lactobacillus* spp. are common bacteria in the cervical and vaginal microbiota (CVM) and are thought to represent a “healthy” cervicovaginal state. Several studies have found an independent association between ethnicity/race and cervical and vaginal microbiota (CVM) composition. Women of sub-Saharan African descent appear to be significantly more likely to have non-*Lactobacillus*-dominated CVM compared to women of European descent. The factors contributing to these differences remain to be fully elucidated. The CVM of Black South African women and factors influencing their CVM remain understudied. In this study, we characterized the cervical microbiota of reproductive-age South African women and assessed the associations of these microbiota with participants’ metadata.

**Methods:**

The cervical microbiota from cervical DNA of 62 reproductive-age women were profiled by Ion Torrent sequencing the V4 hypervariable region of the bacterial 16S ribosomal RNA (rRNA) gene and analyzed with the Quantitative Insights Into Microbial Ecology (QIIME), UPARSE, and metagenomeSeq tools. Associations between cervical microbiota and participants’ metadata were assessed using GraphPad Prism, R packages and an in-house script.

**Results:**

The cervical microbiota clustered into three distinct community state types (CSTs): *Lactobacillus iners*-dominated cervical microbiota (CST I (38.7%, 24/62)), unclassified *Lactobacillus*-dominated cervical microbiota (CST II (4.8%, 3/62)), and diverse cervical microbiota (CST III (56.5%, 35/62)) with an array of heterogeneous bacteria, predominantly the bacterial vaginosis (BV)-associated *Gardnerella*,* Prevotella*, *Sneathia*, and *Shuttleworthia*. CST III was associated with BV (*p* = 0.001). Women in CST I were more likely to be on hormonal contraception, especially progestin-based, compared to women in CST III (odds ratio: 5.2 (95% CI [1.6–17.2]); *p* = 0.005). Women on hormonal contraception had a significantly lower alpha (Shannon indices: 0.9 (0.2–1.9) versus 2.3 (0.6–2.3); *p* = 0.025) and beta (permutational multivariate analysis of variance (PERMANOVA) pseudo-F statistic =4.31, *p* = 0.019) diversity compared to non-users. There was no significant difference in the alpha (Shannon indices: 1.0 (0.3–2.2) versus 1.9 (0.3–2.2); *p* = 0.483) and beta (PERMANOVA pseudo-*F* statistic = 0.89, *p* = 0.373) diversity in women with versus without human papillomavirus infection.

**Conclusions:**

The majority of Black women in our study had non-*Lactobacillus*-dominated cervical microbiota. Additional studies are needed to examine whether such microbiota represent abnormal, intermediate or variant states of health. Lastly, the association of hormonal contraception with *L. iners* dominance requires further in-depth research to confirm this association, determine its biological mechanism and whether it has a beneficial effect on the cervicovaginal health.

## Introduction

It is generally acknowledged that *Lactobacillus* spp., particularly *Lactobacillus crispatus*, *Lactobacillus gasseri*, *Lactobacillus iners*, and *Lactobacillus jensenii*, are common bacteria in the cervical and vaginal microbiota (CVM) (Huang et al., 2015; Ravel et al., 2011; Smith et al., 2012) and are regarded as biomarkers of health (Huang et al., 2015; Petrova et al., 2015; Ravel et al., 2011). Lactobacilli are Gram-positive bacteria (Hillier, 1993) that are thought to have a protective role in preventing genital disease by restricting the growth of non-indigenous organisms, including sexually transmitted infections (STIs) (Borgdorff et al., 2014; Castro et al., 2013; Petrova et al., 2015). *L. gasseri*, for instance, has been associated with rapid clearance of human papillomavirus (HPV) infection (Brotman et al., 2014b). Loss of lactobacilli concomitantly with overgrowth of anaerobic and microaerophilic bacteria, which can be Gram-positive, Gram-negative, and/or Gram-variable bacteria, results in bacterial vaginosis (BV) (Hillier, 1993; Sobel, 2000), the most common form of vaginal disorder among reproductive-age women (Kenyon, Colebunders & Crucitti, 2013). BV has been associated with increased risk of acquisition of a range of STIs such as HPV (Nardis, Mosca & Mastromarino, 2013; Watts et al., 2005) that is associated with cervical neoplasia (Koshiol et al., 2008) and cervical cancer (Bosch et al., 2002). South African women have a high prevalence of BV (31–63%) (Abbai, Reddy & Ramjee, 2016; Kenyon, Colebunders & Crucitti, 2013; Lennard et al., 2018; Onywera et al., 2019) and HPV (16–75%) (Bruni et al., 2019) yet there is paucity of knowledge on the CVM of Black South African women as the existing CVM studies have mostly focussed on women of White, Asian, Hispanic, and African American background.

It is currently known that the composition of the CVM is not only affected by factors ranging from smoking, sexual behaviour, menstrual cycle, and hormonal contraception practices to pregnancy (Borgdorff et al., 2017; Brotman et al., 2014a; Gajer et al., 2012; Romero et al., 2014; Vodstrcil et al., 2017; Wessels et al., 2017), but ethnic/racial background as well (Borgdorff et al., 2017; Fettweis et al., 2014; Ravel et al., 2011; Vodstrcil et al., 2017; Zhou et al., 2007). A large extensive study on 396 asymptomatic reproductive-age multi-ethnic North American women observed different prevalences of *Lactobacillus*-dominated vaginal microbiota among White (89.7%), Asian (80.2%), Hispanic (61.9%), and Black (59.6%) women (Ravel et al., 2011). Most studies have reported that 23–64% of reproductive-age Black women have diverse CVM with low numbers of *Lactobacillus* sp. that are credited for sustaining health (Anahtar et al., 2015; Borgdorff et al., 2014; Dareng et al., 2016; Fettweis et al., 2014; Gautam et al., 2015; Lennard et al., 2018; Onywera et al., 2019; Ravel et al., 2011; Wessels et al., 2017; Zhou et al., 2007). Among women of non-Black ethnicity, this prevalence seldom reaches 40% (Fettweis et al., 2014; Ravel et al., 2011; Zhou et al., 2007). Controversy abounds whether there are actual differences in the CVM composition among ethnic/racial groups or the higher prevalences of diverse non-*Lactobacillus*-dominated CVM in some populations (including Hispanic or Black) may reflect higher rates of asymptomatic BV or STIs (Beamer et al., 2017).

We now know that among the Black South African women with *Lactobacillus*-dominated CVM, a high proportion of them (59–83%) have *L. iners*-dominated CVM (Anahtar et al., 2015; Balle et al., 2018; Lennard et al., 2018; Onywera et al., 2019). This seems contrary to the observation in White women (<45%), where *L. crispatus* is the most predominant *Lactobacillus* spp. (Fettweis et al., 2014; Ravel et al., 2011; Zhou et al., 2007). Of the *Lactobacillus* spp. considered as biomarkers of a healthy cervicovaginal tract (Petrova et al., 2015; Ravel et al., 2011), *L. iners* appears to be the least stable (Gajer et al., 2012; Petrova et al., 2017) and least protective against BV and STIs (Brotman et al., 2014b; Petrova et al., 2017; Van Houdt et al., 2018; Verstraelen et al., 2009). More studies are needed to understand the CVM and factors influencing them. Therefore, owing to the reports on ethnic/racial variations in the CVM and dearth of knowledge about the CVM of Black South African women, we aimed to investigate the baseline structure of cervical microbiota of reproductive-age Black South African women and determine their (microbiota) associations with the participants’ demographic, sociobehavioural, and clinical information.

## Materials and Methods

### Ethics statement

This study was approved by the Human Research Ethics Committee of the University of Cape Town, South Africa (references 258/2006 and 580/2014). All participants provided written informed consent to participate in the study and use of their stored samples for future studies.

### Study population and study design

This was a retrospective cross-sectional study based on data and baseline cervical DNA samples from the HPV Couples Cohort Study (Mbulawa et al., 2009) that examined the transmission of genital HPV among Black heterosexual couples in Gugulethu, Cape Town, South Africa. Details of enrollment, recruitment and sample collection for the HPV transmission study have been described previously (Mbulawa et al., 2009).

In brief, speculum examination was performed and excess mucus around the cervical area was cleared using a filamented swab. This was followed by collection of two cytobrush samples from the cervix. The first sample was for Papanicolaou (Pap) smear or T cell assay. It was collected by inserting the cytobrush into the mouth of the cervix and rotating the cytobrush at 360° thrice. For the Pap smear, the sample was smeared immediately onto the frosted glass slide, quickly fixed using Cytofix spray and then stained with Pap stain. BV was identified on Pap smears by using the Bethesda criteria for reporting cervical/vaginal cytologic diagnoses (Kurman & Solomon, 1994). Smears showing clue cells with coccobacilli (mostly *Gardnerella vaginalis*) and/or any shifts in bacterial flora suggestive of BV (noticeable absence of lactobacilli) on wet microscopy were considered as having findings suggestive of BV. All smears read by cytotechnologists were reviewed. The second sample from the cervix was for HPV genotyping and herpes simplex virus (HSV) testing (and subsequent analyses such as characterization of microbiome). It was collected by inserting a second cytobrush (Digene cervical sampler) into the cervix and rotating it thrice (360°) inside the mouth of the cervix. This sample was then stored in Digene specimen transport medium (Digene Corporation, Gaithersburg, MD, USA) at −80 °C until nucleic acid extraction.

Nucleic acids were extracted from the cervical samples as previously described (2009) (Mbulawa et al., 2009) using the MagNA Pure Compact System and the MagNA Pure Compact Nucleic Acid Isolation kit (Roche Molecular Diagnostics, Mannheim, Germany). HPV typing was also performed as previously documented (Mbulawa et al., 2009) using the Roche Linear Array HPV genotyping test (Roche Molecular Diagnostics, Mannheim, Germany) that detects 37 HPV genotypes. These include 12 oncogenic high-risk, 8 probable oncogenic high-risk, and 17 non-oncogenic low-risk HPV types as listed elsewhere (Mbulawa et al., 2009). Only samples with positive human beta (β)-globin (a housekeeping gene) hybridization results (a measure of sample adequacy) were included in this study. Roche Linear Array HPV genotyping test measures sample adequacy by relying on two endogenous β-globin positive controls (high and low) run concurrently with samples. The primers targeting β-globin are different from those that target the HPV genome (polymorphic L1 region). A valid genotyping result (negative or positive for HPV genotype) is one where both the β-globin probe lines are positive. A result (negative or positive for at least one HPV genotype) is considered invalid if the sample is negative for one or both β-globin control(s). This is suggestive of inadequate cellular material, poor storage and processing (extraction), presence of PCR inhibitors and/or completion with a high titer HPV target.

To be eligible for the present study, only cervical DNA specimens from human immunodeficiency virus (HIV)-seronegative women aged 18-44 years were considered. These samples had to have information on the HPV status and sufficient volume for microbiota analysis (≥15.0 µl of the extracted DNA). Exclusion criteria included being a woman aged <18 or >44 years, self-reported menstruation or pregnancy at the time of sampling, and HIV-seropositivity. Participants’ metadata including demographics, sexual history, smoking, contraceptive use, and clinical characteristics were abstracted from the HPV Couples Cohort Study (Mbulawa et al., 2009).

### Bacterial V4 hypervariable region (16S ribosomal rRNA) library preparation and sequencing

The hypervariable V4 region of the 16S ribosomal rRNA (rRNA) gene was amplified using the universal polymerase chain reaction (PCR) primers 515f (5′-GTGCCAGCMGCCGCG GTAA-3′) and 806r (5′-GGACTACHVGGGTWTCTAAT-3′) (Caporaso et al., 2011). Each PCR contained 1x Ex Taq buffer (Takara Bio Inc., Japan), 0.025 U Ex Taq polymerase, 0.8 mM deoxynucleotide triphosphate (dNTP) mixture, 0.56 mg/ml bovine serum albumin (BSA), 400 nM each primer and 100 ng template. Each sample (and no template PCR control, i.e., nuclease free water) was amplified in 3 replicate reactions. PCR conditions were 98 °C for 2 min, followed by 30 cycles of 98 °C for 20 s, 50 °C for 30 s and 72 °C for 45 s, and a final elongation step at 72 °C for 10 min. The triplicate samples were pooled and purified using the Agencourt AMPure XP system (Beckman Coulter, Germany) according to the manufacturer’s instructions. Amplicon sizes were confirmed by electrophoresis on 1.5% Tris Borate EDTA (TBE) agarose gels and imaging with the ultraviolet transilluminator (UVT) GelDoc-It™ system. The amplicons were quantified using the Quant-iT^^®^^ PicoGreen dsDNA assay (Thermo Fisher Scientific, USA) with FLUOstar OPTIMA Multi-Mode Micro Plate Reader (BMG Labtech, Germany). Sequencing libraries were prepared using the KAPA Library Preparation kit and barcoded Adaptor kits for Ion Torrent™ platforms (KAPA Biosystems, Wilmington, MA, USA). Purified barcoded amplicons were pooled (24 samples per pool) in equimolar amounts and the final library sizes and concentrations assessed on a Bioanalyzer High Sensitivity Chip (Agilent Technologies, Santa Clara, CA, USA). Sequencing was performed on the Ion Torrent Personal Genome Machine (PGM) (Life Technologies, Beverly, MA, USA) at the Central Analytical Facilities (CAF) at Stellenbosch University (Stellenbosch, South Africa).

### Bacterial V4 hypervariable region (16S rRNA) data analysis using bioinformatics tools

The qualities of the raw sequenced reads were visually inspected using FastQC v0.11.2 (Andrews, 2010). Quantitative Insights Into Microbial Ecology (QIIME) v1.8.0 (Caporaso et al., 2010b) with imported UPARSE (usearch7.0.1090) (Edgar, 2013), was used to analyze and interpret the nucleotide sequence data from the cervical microbiota. In the initial sequence pass, reads were quality-filtered and demultiplexed in QIIME using the user-defined parameters in Table S1. Briefly, reads with lengths outside the 200-400-bp range, with a quality score of <25 (sliding window 50) and without barcodes or with any mismatches in the barcode sequences were discarded. A second quality filter was performed in UPARSE with user-defined parameters (Table S1). Sequences were dereplicated followed by abundance sorting and discarding singletons. Operational taxonomic unit (OTU) clustering was performed by UPARSE-OTU method that uses a greedy clustering algorithm, with binning of reads with 97% pairwise identity. This step was performed simultaneously with representative sequence picking and *de novo* chimera filtering. Representative sequences from each unique OTU cluster were picked using abundance algorithm. Additional chimeras were removed by UCHIME algorithm (Edgar et al., 2011). Taxonomy was assigned using the Ribosomal Database Project (RDP) Naïve Bayesian Classifier (Wang et al., 2007), with the Greengenes database (gg13_8 Release) (De Santis et al., 2006). Phylogeny was inferred by aligning representative sequences to Greengenes core set using Python Nearest Alignment Space Termination (PyNAST) (Caporaso et al., 2010a). A phylogenetic tree was built using FastTree. Other parameters used for our analyses are defined in Table S1.

Diversity, rarefaction, and sample ordinations were computed in QIIME. Multiple rarefactions at different sequencing depths were performed, and rarefactions (collector’s) curves plotted to evaluate the completeness of the sampling efforts. Alpha diversity was computed by chao1, observed_species, Shannon, Simpson, and PD_whole_tree metrics. Beta diversity was estimated using weighted and unweighted UniFrac distances, and Bray-Curtis dissimilarity metric. The strength and statistical significance of sample clustering (beta diversity) was computed using permutational multivariate analysis of variance (PERMANOVA) (Anderson, 2001), with 999 permutations. Other biodiversity metrics including Dominance and Shannon Equitability indices were calculated using an in-house script in RStudio v1.1.447 (RStudio Team, 2016). An all-by-all pairwise distance matrix of UniFrac distances were generated and used to hierarchically cluster and ordinate samples. The ordinations were performed using Principal Coordinate Analysis (PCoA).

### Identification and comparison of community state types

Hierarchical clustering and heatmap generation were performed in RStudio v1.1.447 (RStudio Team, 2016). Hierarchical clustering on Bray-Curtis dissimilarity (Vegan package v2.5-5 (Philip, 2003)) was done using the average neighbour algorithm. The heatmap was generated using the heatmap3 v1.1.6 package (Zhao et al., 2014).

### Correlational analyses of 60 cervical bacterial OTUs

To assess bacterial positive and negative relationships, Spearman’s correlation of log_2_-transfomed counts of all OTU pairs were tested on metagenomeSeq v1.12.1 (Paulson et al., 2014; Paulson et al., 2016) in RStudio v1.1.447 (RStudio Team, 2016), and the topmost OTUs (*n* = 60, 28.0%) with the greatest variance displayed on a correlogram.

### Statistical methods

Statistical analyses were carried out using GraphPad Prism v6.01 (San Diego, CA, USA). Mann–Whitney unpaired nonparametric and Chi-square/Fisher’s exact tests (with two-tailed *p*-value) were used to examine the association of continuous and categorical variables with CSTs, as appropriate. The alpha diversity metrics of the three CSTs were compared by Kruskal-Wallis test. Two-group comparison between the alpha diversity of CST, HPV, and BV groups was computed by Mann–Whitney unpaired nonparametric test.

## Results

### Study cohort baseline characteristics

The demographic, sexual and smoking, behavioural and clinical information of the 62 heterosexual Black South African women included in this study are summarized in Table 1. All the women were sexually active, with the majority (72%) of hormonal contraceptives users being on Depo-Provera. HPV and high-risk HPV infections were detected in 37.1% and 29.0% of the women, correspondingly. Thirteen of the women (22.0%) had abnormal cervical cytology. A few women had experienced vaginal discharge (16.1%) and genital ulceration (3.2%) in the last six months. Twenty two women (35.5%) had findings suggestive of BV. A majority of the women (79.0%) had never smoked cigarettes.

### Taxonomic composition of the cervical microbiota

A total of 1,392,562 high-quality non-spurious sequencing reads from 62 samples were included in the final analysis with a median of 16,453 reads per sample (range: 5,343–235,897 per sample). The Ion Torrent PGM raw sequence data and metadata have been deposited in the NCBI Sequence Read Archive (SRA) under BioProject PRJNA472137 (SRP148486) with accession numbers (SRX4103412 –SRX4103416).

A heatmap of the relative abundances of the predominant genera (>0.5% relative abundance) and their respective phyla identified in the cervical microbiota of the 62 women is shown in Fig. S1. Above the 0.5% threshold, only 9 phyla with 43 genera were observed. *Lactobacillus* (46.0% mean relative abundance and 100.0% prevalence), *Gardnerella* (19.5% and 93.5% (58/62)), *Prevotella* (9.7% and 95.2% (59/62)), and *Sneathia* (9.5% and 87.1% (54/62)) were the most predominant genera in the phyla Firmicutes, Actinobacteria, Bacteriodetes, and Fusobacteria, respectively. Other bacteria such as *Dialister* (1.2%), *Atopobium* (0.5%), and *Clostridium* (0.1%) commonly found in women with BV (Srinivasan et al., 2012) were not abundant.

A total of 221 unique OTUs (potential species) were identified in the 62 cervical microbiota and ranged from 16-104 OTUs per sample. The 30 most abundant OTUs represented 97% of all the reads. The most abundant OTU was classified as *L. iners*, representing 40.6% of all the reads. The prevalence and abundance of this OTU together with those of the other most abundant OTUs are shown in Table S2.

*Prevotella* was the most diverse genus with 26 different OTUs identified. Six and eight different OTUs belonging to the genera *Gardnerella* and *Lactobacillus*, respectively, were detected. Apart from *L. iners*, the other *Lactobacillus* OTUs included *Lactobacillus coleohominis*, *Lactobacillus mucosae*, *Lactobacillus ruminis*, and four unclassified *Lactobacillus* spp. Species names could not be assigned to the four *Lactobacillus* OTUs due to insufficient taxonomic discrimination by the V4 hypervariable region of the 16S rRNA gene. Forty seven (75.8%) of the cervical microbiota had at least two *Lactobacillus* spp., but at unequal abundances.

**Table 1:** Baseline demographic, sociobehavioural and clinical information of the 62 heterosexual Black South African women.

Characteristic	All participants
	(*N* = 62)
Age (years)	34.5 (25.8–39.0)
Age at sexual debut (years)^a^	18.0 (17.0–18.8)
Lifetime number of sexual partners^a^	2.0 (2.0–4.0)
Number of sex acts with study partner in last month^a^	2.0 (2.0–4.0)
Current use of hormonal contraceptives* (% (n/N))	
No	55.4 (31/56)
Yes	44.6 (25/56)
Type of hormonal contraceptives (% (n/N))	
Depo-Provera^b^	72.0 (18/25)
Nonrethisterone enanthate^b^	20.0 (5/25)
Oral pills^c^	8.0 (2/25)
HPV (% (n/N))	
Negative	62.9 (39/62)
Positive	37.1 (23/62)
Multiplicity of HPV infection (% (n/N)) among HPV-infected women	
Single infection	65.2 (15/23)
Multiple infections	34.8 (8/23)
Oncogenicity of HPV infection (% (n/N)) among HPV-infected women	
Low-risk genotypes regardless of high-risk genotype	34.8 (8/23)
High-risk genotypes regardless of low-risk genotype	78.3 (18/23)
Both low- and high-risk genotypes	13.0 (3/23)
High-risk (% (n/N))	
Negative	71.0 (44/62)
Positive	29.0 (18/62)
Cervical cytology (% (n/N))	
Normal	78.0 (46/59)
ASCUS	6.8 (4/59)
LSIL	11.9 (7/59)
HSIL	3.4 (2/59)
Experienced vaginal discharge in last 6 months (% (n/N))	
No	83.9 (52/62)
Yes	16.1 (10/62)
Experienced genital ulceration in last 6 months (% (n/N))	
No	96.8 (60/62)
Yes	3.2 (2/62)
Findings suggestive of BV on Papanicolaou smear (% (n/N))	
No	64.5 (40 /62)
Yes	35.5 (22/62)
Cigarette use (% (n/N))	
Never smoked	79.0 (49/62)
Ex-smoker	1.6 (1/62)
Current smoker	19.4 (12/62)

**Notes.**

Abbreviations HPV human papillomavirus ASCUS atypical cells of undetermined significance LSIL low-grade squamous intraepithelial lesion HSIL high-grade squamous intraepithelial lesion BV bacterial vaginosis

Continuous variables are expressed as medians with interquartile ranges (IQRs, at 25^th^ and 75^th^ percentiles).

a Data was not available on the age at sexual debut for two women, lifetime number of sexual partners of two women and number of sexual acts with study partner in the last month of six women.

b Injectable progestin contraceptives.

c The identity of the oral pills (whether oestrogen or progestin or combination) was unknown.

**Figure 1:**
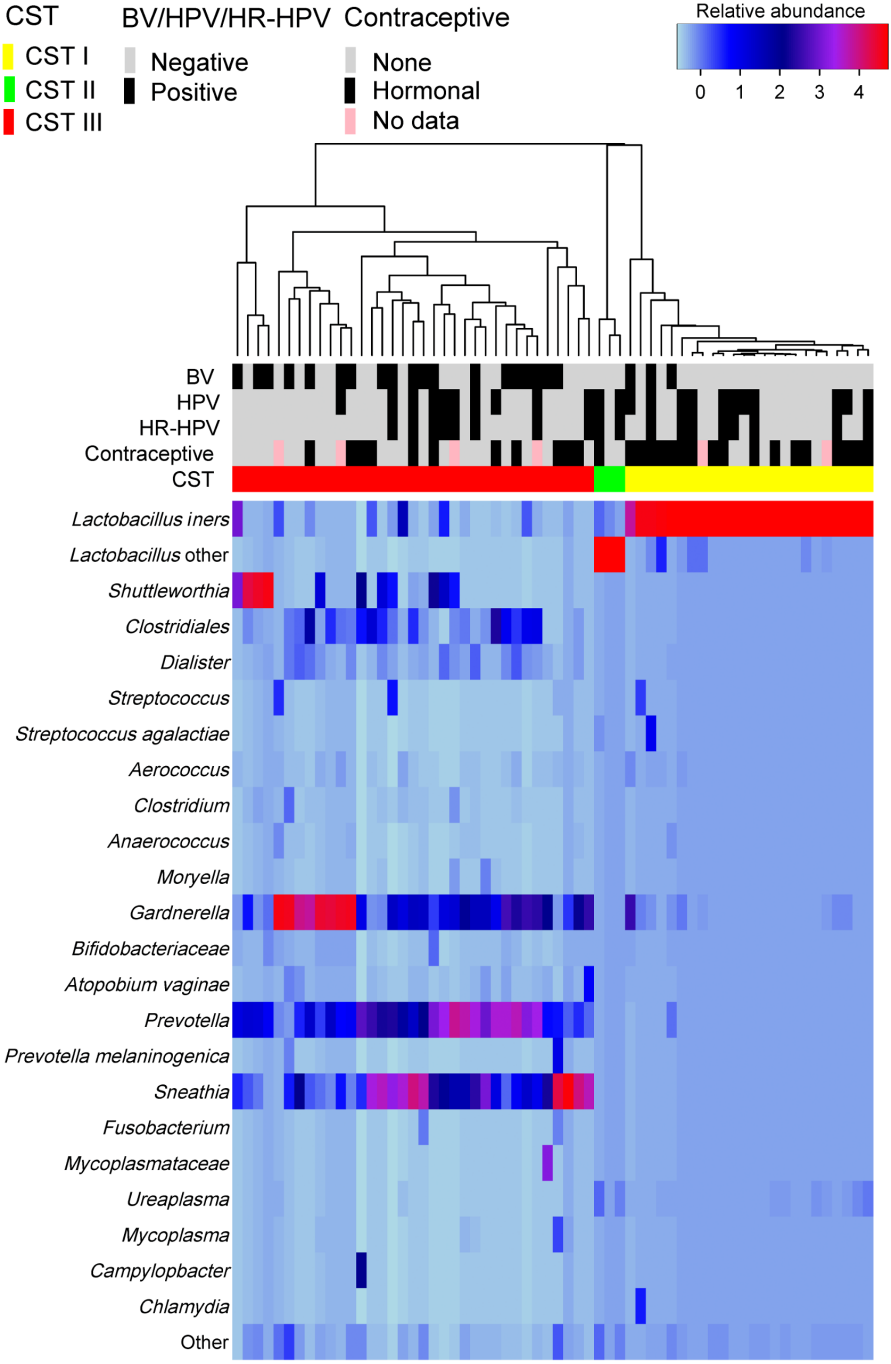
Heatmap of the relative abundances of bacterial taxa in the cervical microbiota of 62 Black South African women. The rows represent the bacterial taxa and columns the samples. The 23 most abundant taxa are displayed, with less abundant taxa grouped as “Other”. The names of the bacteria are presented at the deepest taxonomic level that they were assigned. The dendrogram depicts the average linkage hierarchical clustering of the cervical microbiota based on the Bray-Curtis dissimilarity. The cervical microbiota community state types (CSTs), human papillomavirus (HPV) and high-risk human papillomavirus (HR-HPV) infection status, bacterial vaginosis (BV) findings and contraceptive usage of the women are indicated.

### Characterization of cervical community state types

Hierarchical clustering of the cervical microbiota based on the type and relative abundances of the bacterial taxa identified three distinct community state types, CSTs I–III (Fig. 1). CST I was dominated by *L. iners* and found in 24 women (38.7%). CST II was dominated by an unclassified *Lactobacillus* (*Lactobacillus.4*) and present in only three women (4.8%). CST III was the most common CST occurring in 35 women (56.5%). This CST was characterized by a diverse and complex array of facultative and strictly anaerobic BV-associated bacteria (*Gardnerella*, *Prevotella*, *Sneathia*, *Shuttleworthia*, *Clostridium*, *Atopobium*, *Dialister*, and a consortium of low-abundant bacteria) and very low numbers of *Lactobacillus*, including *L. iners* and three unclassified *Lactobacillus* (*Lactobacillus.1*, *Lactobacillus.3*, and *Lactobacillus.4*). While there was a continuum of relative abundances of the bacterial taxa in CST III, four sub-clusters were evident (Fig. 1), three sub-clusters were dominated by *Shuttleworthia* (*n* = 4 women, 11.4%), *Gardnerella* (*n* = 8, 22.9%), and *Sneathia* ( *n* = 5, 14.3%), and one with mixed taxa (*n* = 18, 51.4%). Next, the metadata for the women in each CST were compared.

### Comparison of the community state types by participants’ metadata

The demographic, sexual, smoking, and clinical characteristics of the women assigned to each of the three CSTs are shown in Table 2. The metadata for women in CST I and CST III were compared, while CST II was excluded from statistical comparisons due to the small sample size.

A significantly greater number of women with cervical microbiota from CST I reported hormonal contraceptive use compared to women with CST III (15/22 (68.2%) versus 9/31 (29%), *p* = 0.005). Findings suggestive of BV on smears were significantly more frequent (*p* = 0.001) in women with CST III than CST I. The other participants’ variables, including HPV status, were not significantly different between the women in CST I and III.

### Comparison of alpha diversity across CSTs, BV, HPV, and hormonal contraceptive use

**Figure 2:**
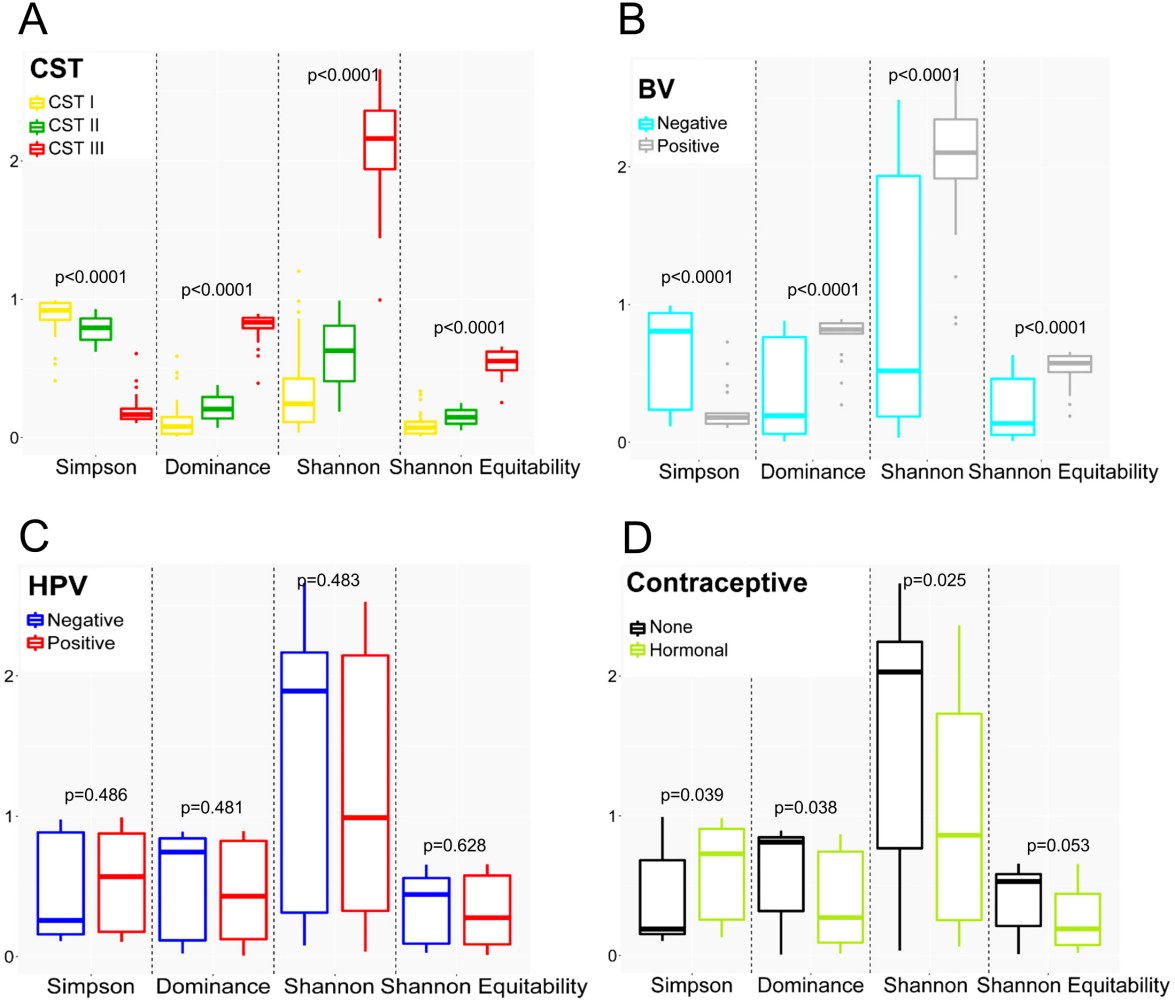
Alpha diversity measures of cervical microbiota. Comparison of the****alpha diversity of the cervical microbiota grouped by: (A) Community state type (CST). (B) Bacterial vaginosis (BV) status. (C) Human papillomavirus (HPV) infection status. (D) Hormonal contraceptive usage. Women with missing information on hormonal contraception were excluded from this analysis. Each box plot is colour-coded according to the type of CST, HPV, BV, and hormonal contraception. In each plot, the box ranges from the first to the third quartile, with the median represented by the horizontal line. The whiskers extend to the smallest and the largest non-outliers and outliers represented by the dots.

Alpha diversity in the cervical microbiota was estimated using a variety of indices, including Simpson, Dominance, Shannon Diversity and Shannon Equitability (Fig. 2). A higher Dominance, Shannon, and Shannon Equitability index value, and lower Simpson index value designates greater alpha diversity. Based on rarefaction curves of alpha diversity metrics (Shannon and Simpson indices, Fig. S2), we chose 5,000 reads per sample as a sufficient subsampling depth to accurately assess microbial diversity.

When grouped by CST (Fig. 2A), CST I (*L. iners*-dominated group) and CST III (diverse group) were significantly different for all the alpha diversity indices (*p* < 0.0001). Thus, bacterial diversity in CST III was significantly greater than CST I.

The diversity of the cervical microbiota of women with findings suggestive of BV was significantly greater than that of women without findings of BV (Shannon index: 2.1 (1.8–2.4) versus 0.5 (0.2–1.9), *p* < 0.0001), Fig. 2B. Alpha diversity was also shown to be significant by Simpson, Dominance, Shannon, and Shannon Equitability metrics.

When grouped by HPV status (Fig. 2C), no significant difference in alpha diversity was observed between the HPV-negative and HPV-positive groups, with Shannon index of 1.9 (0.3–2.2) and 1.0 (0.3–2.2), *p* = 0.483, respectively. Alpha diversity was also shown to be significant by Simpson, Dominance, Shannon, and Shannon Equitability metrics.

**Table 2:** Clinical, demographic, sociobehavioural, and microbiological characteristics of the women by cervical community state type.

Characteristic	CST I	CST II	CST III	*p*-value^a^
	(*N* = 24, 38.7%)	(*N* = 3, 4.8%)	(*N* = 35, 56.5%)	
Age (years)	30.5 (22.3–37.8)	31.0 (30.0–32.0)	35.0 (28.0–40.0)	0.203
HPV infection (% (n/N))				
Any HPV type	45.8 (11/24)	66.7 (2/3)	28.6 (10/35)	0.174
Any high-risk type	33.3 (8/24)	66.7 (2/3)	22.9 (8/35)	0.374
Single infection	25.0 (6/24)	66.7 (2/3)	20.0 (7/35)	0.301
Multiple infection	20.8 (5/24)	0.0 (0/3)	8.6 (3/35)	
HPV status at 6 month visit^e^ (% (n/N))				
Negative	42.9 (6/14)	0.0 (0/2)	56.0 (14/25)	0.073
Acquired	0.0 (0 /14)	0.0 (0/2)	16.0 (4/25)	
Cleared	7.1 (1/14)	50.0 (1/2)	16.0 (4/25)	
Persisted	21.4 (3/14)	50.0 (1/2)	8.0 (2/25)	
Age at sexual debut (years)^d^	17.0 (16.0–18.0)	18.0 (18.0–19.0)	18.0 (17.0–19.0)	0.289
Lifetime number of sexual partners	2.0 (2.0–3.0)	2.0 (2.0–6.0)	2.0 (2.0–4.0)	0.711
Number of sex acts with study partner in last month^d^	2.0 (2.0–4.0)	2.0 (2.0–4.0)	2.0 (2.0–4.0)	0.445
Currently on hormonal contraceptives (% (n/N))	68.2 (15/22)	33.3 (1/3)	29.0 (9/31)	0.005
Depo-Provera^f^	50.0 (11/22)	33.3 (1/3)	19.4 (6/31)	0.019
Nonrethisterone enanthate^f^	18.2 (4/22)	0.0 (0/3)	3.2 (1/31)	0.147
Oral pills^g^	0.0 (0/22)	0.0 (0/3)	6.5 (2/31)	0.505
Cervical cytology (% (n/N))				
Normal	75.0 (18/24)	33.3 (1/3)	84.4 (27/32)	0.536
ASCUS	4.2 (1/24)	33.3 (1/3)	6.3 (2/32)	
LSIL	16.7 (4/24)	0.0 (0/3)	9.4 (3/32)	
HSIL	4.2 (1/24)	33.3 (1/3)	0.0 (0/32)	
Experienced vaginal discharge in last 6 months (% (n/N))	20.8 (5/24)	0.0 (0/3)	14.3 (5/35)	0.726
Experienced genital ulceration in last 6 months (% (n/N))	4.2 (1/24)	0.0 (0/3)	2.9 (1)	1.000
Positive for findings suggestive of BV on Papanicolaou smear (% (n/N))	12.5 (3/24)	0.0 (0/3)	54.3 (19/35)	0.001
Cigarette use (% (n/N))				
Never smoked	87.5 (21/24)	100.0 (3/3)	71.4 (25/35)	0.091
Ex-smoker	4.2 (1/24)	0.0 (0/3)	0.0 (0/35)	
Current smoker	8.3 (2/24)	0.0 (0/3)	28.6 (10/35)	
Prevalence (and mean relative abundance) of descriptive microbiological feature (bacteria)^b^				
*L. iners*	100.0 (91.7)	100.0 (3.2)	100.0 (2.9)	<0.0001
*L. coleohominis*	12.5 (<0.1)	0.0 (0.0)	0.0 (0.0)	0.062
*L. mucosae*	4.2 (<0.1)	0.0 (0.0)	0.0 (0.0)	0.407
*L. ruminis*	12.5 (<0.1)	0.0 (0.0)	0.0 (0.0)	0.062
*Lactobacillus.1*	100.0 (0.3)	100.0 (0.1)	14.3 (<0.1)	<0.000^c^
*Lactobacillus.2*	37.5 (0.1)	33.3 (0.5)	0.0 (0.0)	0.0001^c^
*Lactobacillus.3*	45.8 (1.1)	33.3 (<0.1)	5.7 (<0.1)	<0.0001^c^
*Lactobacillus.4*	37.5 (0.1)	100.0 (87.8)	45.7 (<0.1)	0.648
*Clostridiales*	29.2 (<0.1)	33.3 (<0.1)	94.3 (6.0)	<0.0001^c^
*Dialister*	33.3 (<0.1)	33.3 (<0.1)	100.0 (2.3)	<0.0001^c^
*Gardnerella*	91.6 (2.5)	33.3 (<0.1)	100.0 (25.3)	<0.0001
*Prevotella*	75.0 (0.3)	100.0 (0.2)	100.0 (22.5)	<0.0001^c^
*Shuttleworthia*	33.3 (<0.1)	66.7 (<0.1)	54.3 (9.7)	<0.0001
*Sneathia*	75.0 (<0.1)	66.7 (<0.1)	97.1 (22.7)	<0.0001^c^
Alpha diversity				
Simpson index	0.9 (0.8–1.0)	0.8 (0.6–0.9)	0.2 (0.1–0.2)	<0.0001
Dominance index	0.1 (0.0–0.2)	0.2 (0.1–0.4)	0.8 (0.8–0.9)	<0.0001
Shannon index	0.2 (0.1–0.5)	0.6 (0.2–1.0)	2.2 (1.9–2.4)	<0.0001
Shannon Equitability index	0.1 (0.0–0.1)	0.1 (0.1–0.3)	0.6 (0.5–0.6)	<0.0001

**Notes.**

Abbreviations HPV human papillomavirus ASCUS atypical cells of undetermined significance LSIL low-grade squamous intraepithelial lesion HSIL high-grade squamous intraepithelial lesion BV bacterial vaginosis CST community state type

a *p*-values are shown for the comparison of CST I and CST III. Associations of continuous variables (expressed as medians with interquartile ranges (IQRs, at 25^th^ and 75^th^ percentiles)) and categorical variables were computed by Mann-Whitney unpaired and Chi-square/Fishers exact tests, respectively. CST II was excluded from the statistical analyses due to the low sample number (*n* = 3; 4.8%). Significant *p*-values (<0.05) are shown in bold.

b *p*-values are for differences in relative abundances.

c Prevalences were significantly different (*Lactobacillus.1* (*p* < 0.0001), *Lactobacillus.2* (*p* = 0.0001), *Lactobacillus.3* (*p* = 0.0003), *Clostridiales* (*p* < 0.0001), *Dialister* (*p* < 0.0001), *Prevotella* (*p* = 0.003), *Sneathia* (*p* = 0.015)).

d Data was not available on the age at sexual debut for two women (one CS T I and one CST III) and number of sexual acts wit h study partner in the last month for two women (one CST I and one CST III).

e Data was available for 41 women only. Five of these women (four CST I and one CST III) had complex HPV infection patterns, which were a combination of either cleared and acquired (three women) or cleared and persistent (two women) infections with specific HPV genotypes.

f Injectable progestin contraceptives.

g The identity of the oral pills (whether oestrogen or progestin or combination) was unknown.

Lastly, hormonal contraceptive users (Shannon index: 0.9 (0.2–1.9)) had a lower microbial diversity than non-users (2.3 (0.6–2.3)), *p* = 0.025 (Fig. 2D). When stratified by the type of contraceptive used, the diversities based on Shannon index were significantly different (*p* = 0.028); microbial diversity of Depo-Provera (0.7 (0.2–1.7)) and norethisterone enanthate (0.5 (0.2–1.4)) was significantly lower than that of non-users (2.0 (0.6–2.3)) (Fig. S3).

### Comparison of beta diversity across CSTs, BV, HPV, and hormonal contraceptive use

**Figure 3:**
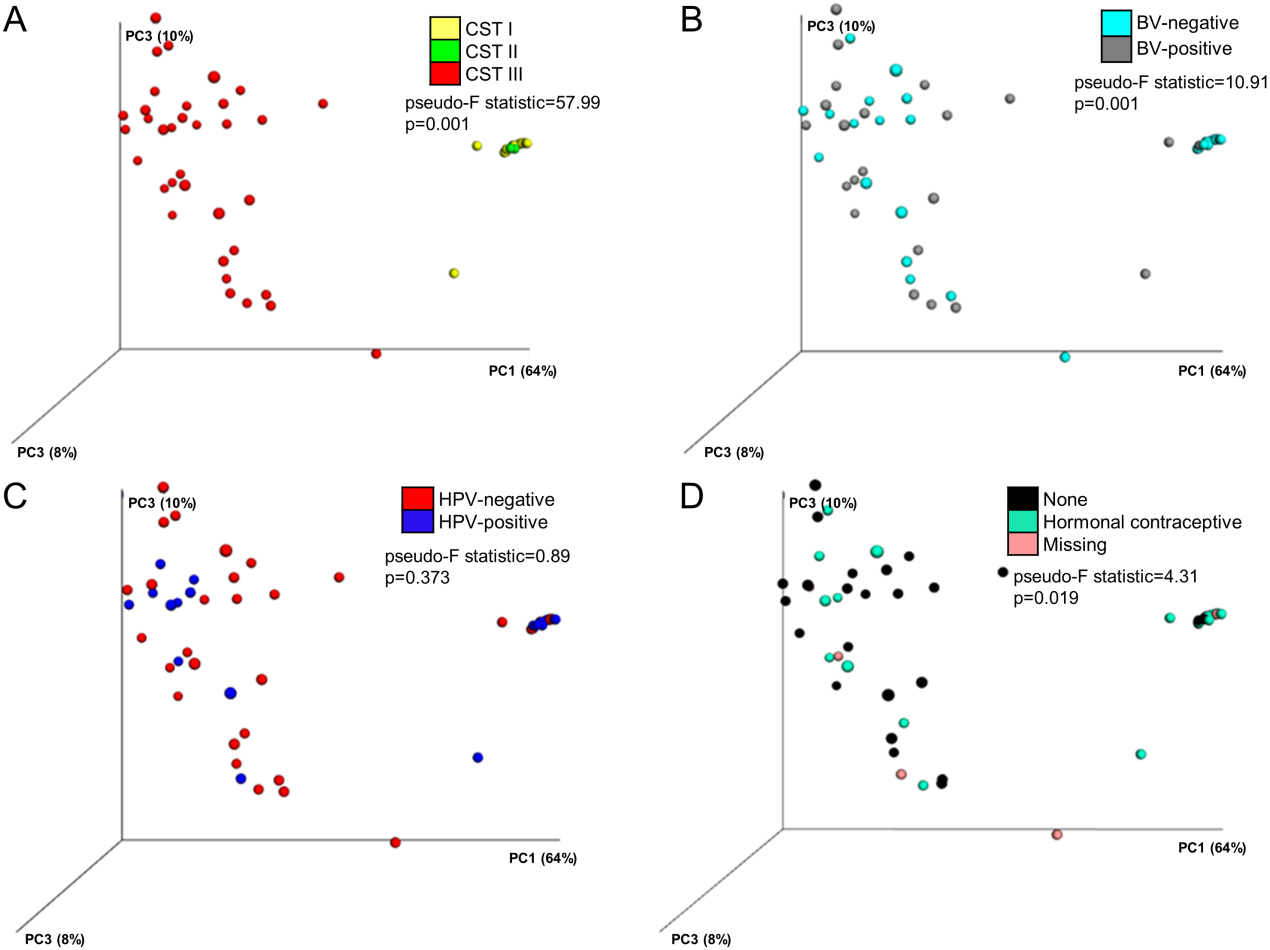
Beta diversity of the cervical microbiota. Principal Coordinates Analysis (PCoA) plots of the weighted UniFrac distances of the cervical microbiota coloured according to:****(A) Community state type (CST). (B) Bacterial vaginosis (BV) status. (C) Human papillomavirus (HPV) infection status. (D) Hormonal contraception usage. The first three principal coordinate (PC) axes and the percentage variation explained by each (PC1: 64%, PC2: 10%, and PC3: 8%) are shown. Each solid point represents a bacterial community.

Beta diversity analysis (PCoA of weighted UniFrac distances) of the 62 samples showed that each of the established CST (I-III) represented a highly distinct bacterial community, *p* = 0.001 (Fig. 3A). This result was supported by the Jackknife replicates that were used to estimate the uncertainty in PCoA plots and hierarchical clustering of the cervical microbiota. The majority of the samples with *Lactobacillus*-dominated cervical microbiota (CST I and CST II, 25/27, 92.6%) exclusively clustered together in the upper right quadrant (Fig. 3A). Two samples from CST I did not cluster with this group likely due to presence of other bacterial taxa, e.g., *Gardnerella*, *Prevotella*, and *Aerococcus*, in these cervical microbiota. These samples were from women with BV.

Further, beta diversity analysis showed that the clustering of the samples was dependent on the findings suggestive of BV, *p* = 0.001 (Fig. 3B). The 25 samples that clustered together in the upper right quadrant consisted mostly of women without findings suggestive BV (22/25, 88.0%). Samples from women with findings suggestive of BV were spread over a greater area in the plot due to their high and varying bacterial diversity.

The weighted UniFrac distances of the cervical microbiota showed that there was no apparent influence of HPV infection on beta diversity, *p* = 0.373 (Fig. 3C). Of the samples that clustered together, 48.0% (12/25) were HPV-positive.

The majority of the samples from women on hormonal contraception (16/25, 64.0%), mostly Depo-Provera (12/18, 75.0%), clustered together in the upper right quadrant, *p* = 0.019 (Fig. 3D). Whereas 80.0% (4/5) of the samples from women on norethisterone enanthate clustered together with the 75.0% samples from women on Depo-Provera, all the samples from women on oral contraceptives (2/2, 100.0%) did not (*p* = 0.018).

### Co-occurrence and co-exclusion patterns of cervical bacterial OTUs

**Figure 4:**
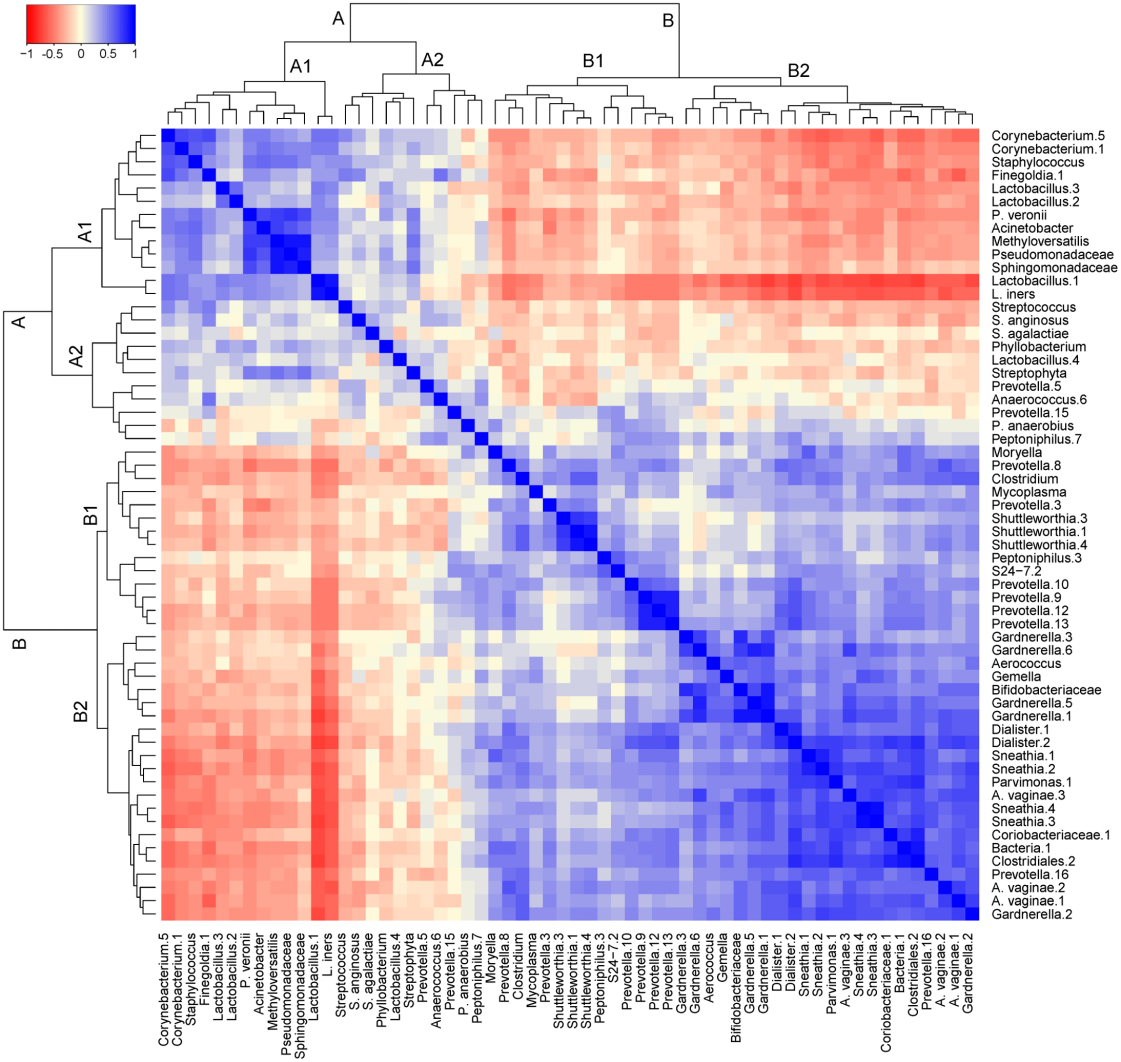
Correlogram of 60 cervical bacterial OTUs showing co-occurrence and co-exclusion patterns. Spearman’s rank correlations between OTU counts were calculated in metagenomeSeq and the samples clustered. The correlation coefficients range from −1 (red; incompatibilities, co-exclusions, or oppositional interactions) to +1 (blue; symbiotic, mutualistic, or co-occurrence interactions.

Pairwise correlations were calculated for all pairs of OTUs identified in the cervical microbiota. A correlation matrix of the pairwise correlations between the 60 most abundant cervical bacterial OTUs is shown in Fig. 4.

From the dendrogram, two major bacterial correlation clusters, Cluster-A (mostly with OTUs classified as *Lactobacillus* and *Streptococcus*) and Cluster-B (mostly with BV-associated bacteria) were observed. Each of these clusters had two sub-clusters: Cluster-A1 and Cluster-A2 for Cluster-A, and Cluster-B1 and Cluster-B2 for Cluster-B. OTUs in Cluster-A had an inverse correlation with OTUs in Cluster-B. For example, *Lactobacillus* OTUs (in Cluster-A) had negative correlations with *Gardnerella* and *Prevotella* OTUs (in Cluster-B). OTUs in the same Cluster-A1 had stronger positive correlations with one another than with OTUs in another sub-cluster from the same cluster (e.g., Cluster-A2). There was some overlap in the interaction of bacteria in Cluster-A2 and Cluster-B1. Interactions between these sub-clusters appeared very low to moderate. We noted that strong positive correlations were very common between phylogenetically related bacterial OTUs, e.g., *Lactobacillus* spp., but the extent of these interactions varied.

## Discussion

Using a culture-independent analysis of cervical microbiota of 62 reproductive HIV-seronegative Black women, we identified three CSTs (CST I: dominated by *L. iners*, CST II: dominated by an unclassified *Lactobacillus* OTU, and CST III: diverse and heterogeneous cervical microbiota) and found a positive association of hormonal contraception (mostly progestin-based) with CST I.

Among the women with *Lactobacillus* dominance in our cohort, CST I (*L. iners*-dominated) was the most prevalent (89%). *L. iners*-dominated CVM are the most prevalent CSTs with *Lactobacillus* dominance among women of African ancestry (Borgdorff et al., 2017; Fettweis et al., 2014; Ravel et al., 2011; Zhou et al., 2007), including those in sub-Saharan Africa (prevalence: 42–88%) (Anahtar et al., 2015; Balle et al., 2018; Borgdorff et al., 2014; Dareng et al., 2016; Gautam et al., 2015; Lennard et al., 2018; Onywera et al., 2019; Wessels et al., 2017). The variations in the prevalence of *L. iners* predominance (42–89%) could be attributed to the differences in anatomical sample type (whether cervical or vaginal) (Balle et al., 2018; Huang et al., 2015; Kim et al., 2009), sampling technique (Kim et al., 2009), behaviour (Borgdorff et al., 2017; Brotman et al., 2014a; Vodstrcil et al., 2017; Wessels et al., 2017), and genetics within the Black population (Borgdorff et al., 2017; Tishkoff & Williams, 2002). For instance, a study on a Kenyan cohort found that non-sex workers were more likely to have *L. iners*-dominated microbiota compared to female sex workers, FSWs (women who engaged in high-risk sexual behaviour) (Wessels et al., 2017). Overall, all the women (100.0%) in our study had detectable *L. iners*. This finding is qualitatively congruent with those of culture-independent studies (Borgdorff et al., 2014; Ravel et al., 2011) but contrasts those reported in cultivation studies on premenopausal South African women (36–75%) (Damelin et al., 2011; Pendharkar et al., 2013). This dissimilarity could be due to difficulty in culturing *L. iners* (Lamont et al., 2011; Srinivasan et al., 2016), the higher sensitivity of deep sequencing technology (Fettweis et al., 2012; Nielsen et al., 2007), and/or ability of DNA-based identification methods (e.g., PCR) to detect relic DNA (from non-viable or dead bacteria) besides DNA from viable bacteria (Nielsen et al., 2007). It is believed that the detectability of *Lactobacillus* spp. in all the cervical microbiota attests that the production of lactic acid is conceivably conserved in all microbiota (Ravel et al., 2011), thus pinpointing it as the core acidifier (Boskey et al., 1999).

The role of *L. iners* in the cervicovaginal health is however unclear (Petrova et al., 2017). Unlike many *Lactobacillus* spp., *L. iners* can occur with BV-associated bacteria (Borgdorff et al., 2017; Gautam et al., 2015; Petrova et al., 2017; Srinivasan et al., 2012) and can at times enhance their adhesion to cervical epithelium (Castro et al., 2013). Additionally, it has been consistently isolated from women with and without vaginal syndromes, intermediate flora (Damelin et al., 2011; Pendharkar et al., 2013; Petrova et al., 2017; Srinivasan et al., 2016; Zozaya-Hinchliffe et al., 2010), or women with CSTs transitioning to healthy or dysbiotic states (Gajer et al., 2012; Petrova et al., 2017). Growth of *L. iners* in BV-associated environment could be due to its inefficient colonization resistance to opportunistic and pathogenic bacteria (Pendharkar et al., 2013; Zhou et al., 2007) or better tolerant and survival phenotypes even in perturbed milieus (Pendharkar et al., 2013; Zozaya-Hinchliffe et al., 2010). There is compelling omics evidence supporting the second explanation. Genomics have suggested that *L. iners* underwent rapid evolutionary events that endowed it with competitive and specialized adaptation capabilities even in dysbiotic milieu (Macklaim et al., 2010). Meta-transcriptomics have strengthened these facts, demonstrating that *L. iners* is able to differentially express over 10% of its genome in order to survive in dysbiotic state (Macklaim et al., 2013). *L .iners* can predispose women to an aberrant microbiota or BV (Verstraelen et al., 2009) and has been associated with STIs (Borgdorff et al., 2014; Brotman et al., 2014b; Van Houdt et al., 2018). In contrast to this detrimental outcome, *L. iners* can interfere with *G. vaginalis* biofilm assembly (Saunders et al., 2007), thereby restoring a healthy CVM. More exploratory studies are therefore needed to characterize cervicovaginal *L. iners* since it is currently believed that it has clonal variants that may have different roles in health, dysbiosis, and disease (Petrova et al., 2017).

In our study, about 5% of the women had CVM dominated by an unclassified *Lactobacillus* sp. (CST II). Similar to a previous study that used a similar methodology (Roesch et al., 2017), the V4 hypervariable region of the 16S rRNA gene did not allow us to achieve a deeper taxonomic discrimination of the *Lactobacillus* in CST II. Therefore, we could not ascertain whether the low prevalent CST II was one of the commonly established CST with *Lactobacillus* (*L. crispatus*, *L. gasseri*, or *L. jensenii*) dominance as found elsewhere (Ravel et al., 2011). Generally, *L. crispatus*, *L. gasseri*, and *L. jensenii* are often less common and less abundant in Black women compared to White women (Balle et al., 2018; Borgdorff et al., 2014; Fettweis et al., 2014; Lennard et al., 2018; Ravel et al., 2011; Zhou et al., 2007), with *L. crispatus* recently found to be less abundant in cervical samples (similar to our study’s) compared to lateral vaginal wall samples (Balle et al., 2018). While we detected other *Lactobacillus* spp. such as *L. coleohominis*, *L. mucosae*, and *L. ruminis* that have been uncovered from premenopausal South African women (Damelin et al., 2011; Pendharkar et al., 2013), we also confirmed that microbiota with approximately equal dominance of two or more *Lactobacillus* spp. are absent or underrepresented in Black women (Zhou et al., 2007).

The prevalence of diverse and heterogeneous group, CST III (57%), was higher than has been documented in non-Black women (Borgdorff et al., 2017; Fettweis et al., 2014; Ravel et al., 2011; Zhou et al., 2007) and intermediate to what has been recently reported among Black South African women (47–64%) (Anahtar et al., 2015; Lennard et al., 2018; Onywera et al., 2019). CST III exhibited four intracluster variations: CST III-*Shuttleworthia*, CST III-*Gardnerella*, CST III-*Sneathia*, and CST III-mixed, which lacked a clear dominance. The observation of *Shuttleworthia* should be treated with scepticism, it is perhaps a misclassification of BV-associated bacterium-1 (BVAB-1) (Oakley et al., 2008). *Gardnerella*-dominated microbiota have been identified in African American (Zhou et al., 2007), Black South African (Anahtar et al., 2015), African Surinamese, and Ghanaian (Borgdorff et al., 2017) women. CST III was associated with BV, thus, confirming earlier findings (Onywera et al., 2019; Ravel et al., 2011; Srinivasan et al., 2012; Zozaya-Hinchliffe et al., 2010). It is important to point out that diversity of CVM as observed by 16S rRNA sequencing may not always be associated with BV as diagnosed by laboratory tests. This was corroborated in a study by Wessels and colleagues (2017) that found that a majority of FSW (74%) without BV (Nugent score: 0–3) had highly diverse vaginal communities (as captured by 16S rRNA sequencing) (Wessels et al., 2017).

While this study was not specifically designed to assess the impact of hormonal contraceptives on the cervical microbiota, we did observe that hormonal contraceptive use (mostly progestin: at least 92%) was correlated with *L. iners* dominance of the cervical microbiota in reproductive-age Black South African women. At the moment, there is a wide heterogeneity of results regarding the effect of hormonal contraception on the composition and diversity of the CVM. Such results include (i) increased detectability of *Lactobacillus fermentum* among Pakistani woman on oral contraception (Kazi, Saleem & Kazi, 2012), (ii) higher abundances of *Lactobacillus* spp. (*L. crispatus* and *L. jensenii*) and *L. iners* among US women on oral and progestin-based contraception, respectively (Brooks et al., 2017), (iii) no effect on *L. iners* quantities, reduced total bacterial load and *G. vaginalis* following progestin-based initiation among Kenyan women (Roxby et al., 2016), (iv) decreased levels of *L. iners* but not of the other beneficial *Lactobacillus* spp. (*L. crispatus*, *L. gasseri*, and *L. jensenii*) in response to progestin-based contraception among Zimbabwean women (Achilles et al., 2018), (v) reduced lactobacilli following long-term use of progestin-based contraception among US women (Mitchell et al., 2014), and so on. We further noted that women using hormonal contraceptives (mostly progestin) had a lower bacterial (alpha) diversity relative to non-users. Previous studies have found either no discernible (Birse et al., 2017) or positive associations between progestin-based contraception and the level of bacterial diversity (Brooks et al., 2017; Jespers et al., 2017; Yang et al., 2019). The differential effects of hormonal contraception, particularly progestin-based, could be governed by host genetics (Yang et al., 2019) and differences in study methodologies. Although current literature suggests that hormonal contraceptives (oestrogen- and/or progestin-based) reduces the risk of BV-associated bacteria (Brooks et al., 2017), BV-like microbiota (Borgdorff et al., 2017) and BV (Abbai, Reddy & Ramjee, 2016; Vodstrcil et al., 2013), with progestin-based contraceptives reducing BV risk by 18–30% (Van de Wijgert et al., 2013), more studies are still needed to fully understand the microbiological consequences of progestin-induced amenorrhea and systemic hypoestrogenism in the context of reproductive health (Miller et al., 2000).

In the present study, except for BV and hormonal contraception, we did not find any association of cervical microbiota with the other participant variables. For example, we observed no significant difference in prevalent HPV between *L. iners* dominated and diverse microbiota, a finding that mirrors previous studies (Borgdorff et al., 2014; Brotman et al., 2014b; Onywera et al., 2019). There may be no association or, more likely, this was due to methodological shortcomings in our study, including the relatively small sample size.

Finally, our results on correlational analyses provided evidence for synergism (co-occurrence) and antagonism (co-exclusion) of the cervicovaginal bacterial communities. These ecological patterns have been elucidated to act concurrently to structure the microbiota compositions (Zelezniak et al., 2015). Positive correlations suggest niche sharing, similar nutrient requirements and or metabolic co-dependencies. Co-occurrence patterns, for example, of *Gardnerella* with *Prevotella* and other BV-associated bacteria parallels previous findings (Ravel et al., 2011). Moreover, phylogenetically related bacterial OTUs such as *Prevotella* spp. had strong positive correlations, which may be due to the high-level of resource overlap (Zelezniak et al., 2015). Variations in positive correlations of more closely phylogenetically-related bacteria like the different *Atopobium* spp., or *Gardnerella* spp., illuminates the existence of diverse bacterial genetic profiles at species level (De Backer et al., 2006; Eren et al., 2011), plausibly with different virulent competencies. Strains of *Atopobium* spp. and *Gardnerella* spp. have been demonstrated to have dissimilar phenotypic behaviours in cervicovaginal health and disease (De Backer et al., 2006; Swidsinski et al., 2010). Inverse correlations such as those observed between *Lactobacillus* and BV-associated bacterial OTUs in our study and others (Anahtar et al., 2015; Castro et al., 2013; Ravel et al., 2011; Srinivasan et al., 2012; Srinivasan et al., 2010), are indications for niche filtering and/or competition for growth nutrients (Srinivasan et al., 2012; Zelezniak et al., 2015). As stated by Ravel and co-workers (2011), the precise relevance of these bacterial positive and negative interactions remains undefined; therefore, subject to further investigations.

Even though the present study broadens our knowledge about the ethnic differences in the composition of cervical microbiota and associations of particular microbiota with clinical and behavioural characteristics, a few limitations arising from this retrospective cross-sectional study should be noted. First, while we found an association between hormonal contraception (mostly progestin) and *L. iners* dominance, we did not adjust our analysis for potential confounders such as age, time of sampling (with regards to hormonal/menstrual cycle stage and length of time since last dose of hormonal contraception), length of time on hormonal contraception, sexual behaviour, STIs, grouping all forms of hormonal contraceptives together (as they might be exerting different effects on cervical microbiota), and vaginal disorders (e.g., BV and aerobic vaginitis) to name a few. Secondly, small sample size in some groups limited additional comparisons. In addition, we might have inadequately diagnosed BV since we used cervical samples and a non-standard approach (Pap smear) to diagnose BV. It is believed that BV is best diagnosed with vaginal instead of cervical samples (Hillier, 1993). Pap smear has been demonstrated not to perform well (specificity: 93–94% and sensitivity: 43–49%) when compared to Gram stain diagnosis (Greene, Kuehl & Allen, 2000; Tokyol et al., 2004). Some investigators consider techniques that primarily rely on the presence of *Gardnerella* to diagnose BV to be inappropriate. (Dols et al., 2011). However, the high specificity of Pap smear suggests that “it may be an adequate diagnostic criterion when it is positive” (Tokyol et al., 2004). Lastly, failure to confidently assign species names to some bacteria impeded accurate comparison of our results to other published studies. Future studies would be of more benefit if all these limitations are addressed.

## Conclusions

A majority of the reproductive-age HIV-seronegative Black South African women (57%) had cervical microbiota not dominated by *Lactobacillus*, the bacteria assumed to constitute a healthy cervical microbiota. These cervical microbiota were associated with findings suggestive of BV. It has been speculated that such cervical microbiota may be a contributing factor to the high burden of HIV and HPV infections among Black women (Zhou et al., 2007). Not all women (46%) with non-*Lactobacillus*-dominated cervical microbiota had findings suggestive of BV. Hence, additional studies are needed to examine whether these cervical microbiota signify abnormal, intermediate or variant states of health in Black women. The association of hormonal contraceptive (mostly progestin) use with *L. iners* dominance merits further investigation as there is still paucity of studies, uncertainty and controversy surrounding this topic.

##  Supplemental Information

10.7717/peerj.7488/supp-1 Figure S1 Relative abundances of bacterial genera in the cervical microbiota of the 62 women Only bacteria that occurred at .0.5% relative abundances are shown. Each dot on the x-axis represents a participant. The vertical (dashed) line stratifies the heatmap according to HPV status (left –HPV-negative and right –HPV-positive). The horizontal (solid) lines separate the different phyla, as indicated on the right.

10.7717/peerj.7488/supp-2 Figure S2 Rarefaction plots of the 62 cervical microbiota Rarefaction plots based on: (A) Shannon diversity index (a quantitative measure of community richness). (B) Simpson diversity index (a qualitative and quantitative measure of community richness). The alpha diversity was assessed at different sequencing depths. Each coloured-curve represents one of the 62 samples.

10.7717/peerj.7488/supp-3 Figure S3 Alpha diversity grouped by hormonal contraception status and type of contraceptive Each box plot is colour-coded according to the type of hormonal contraception. In each plot, the box ranges from the first to the third quartile, with the median represented by the line that divides the box into two. The whiskers extend to adjacent values within the lower and upper quartiles. Outliers are represented by the dots. Hormonal contraceptive users are categorized according to the type of contraceptive: oral (oral pills) or injectable (Depo: Depo-Provera and Neten: norethisterone enanthate) contraceptives. Besides being displayed in this figure, women in the oral pills group were not included in the calculation of the alpha diversity due to the small sample size in this group (*n* = 2).

10.7717/peerj.7488/supp-4 Table S1 QIIME and UPARSE selected parameters for cervical microbiota analyses

10.7717/peerj.7488/supp-5 Table S2 Prevalence and mean relative abundance of the most abundant bacterial OTU Abbreviation: OTU operational taxonomic unit.

## Additional Information and Declarations

### Data Availability

The following information was supplied regarding data availability: Data is available at NCBI SRA, accession numbers: SRX4103412, SRX4103413, SRX4103414, SRX4103415, SRX4103416, PRJNA472137.
