# Supplementary figures and images for: Factors associated with the composition and diversity of the cervical microbiota of reproductive-age Black South African women: a retrospective cross-sectional study
Source: PeerJ. 2019 Aug 15;7:e7488. doi: 10.7717/peerj.7488 (PMC6698374; doi:10.7717/peerj.7488)

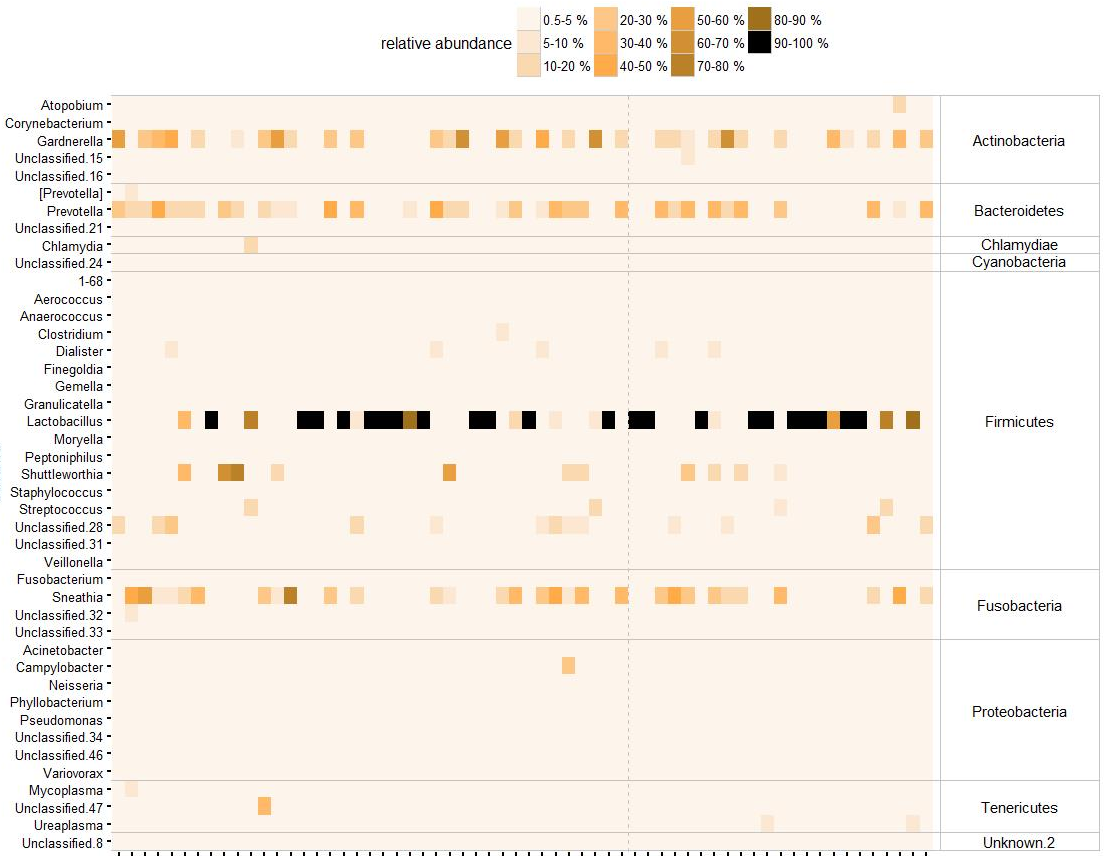

Supplement: Figure S1 — Only bacteria that occurred at .0.5% relative abundances are shown. Each dot on the x-axis represents a participant. The vertical (dashed) line stratifies the heatmap according to HPV status (left –HPV-negative and right –HPV-positive). The horizontal (solid) lines separate the different phyla, as indicated on the right. [file peerj-07-7488-s001.png]

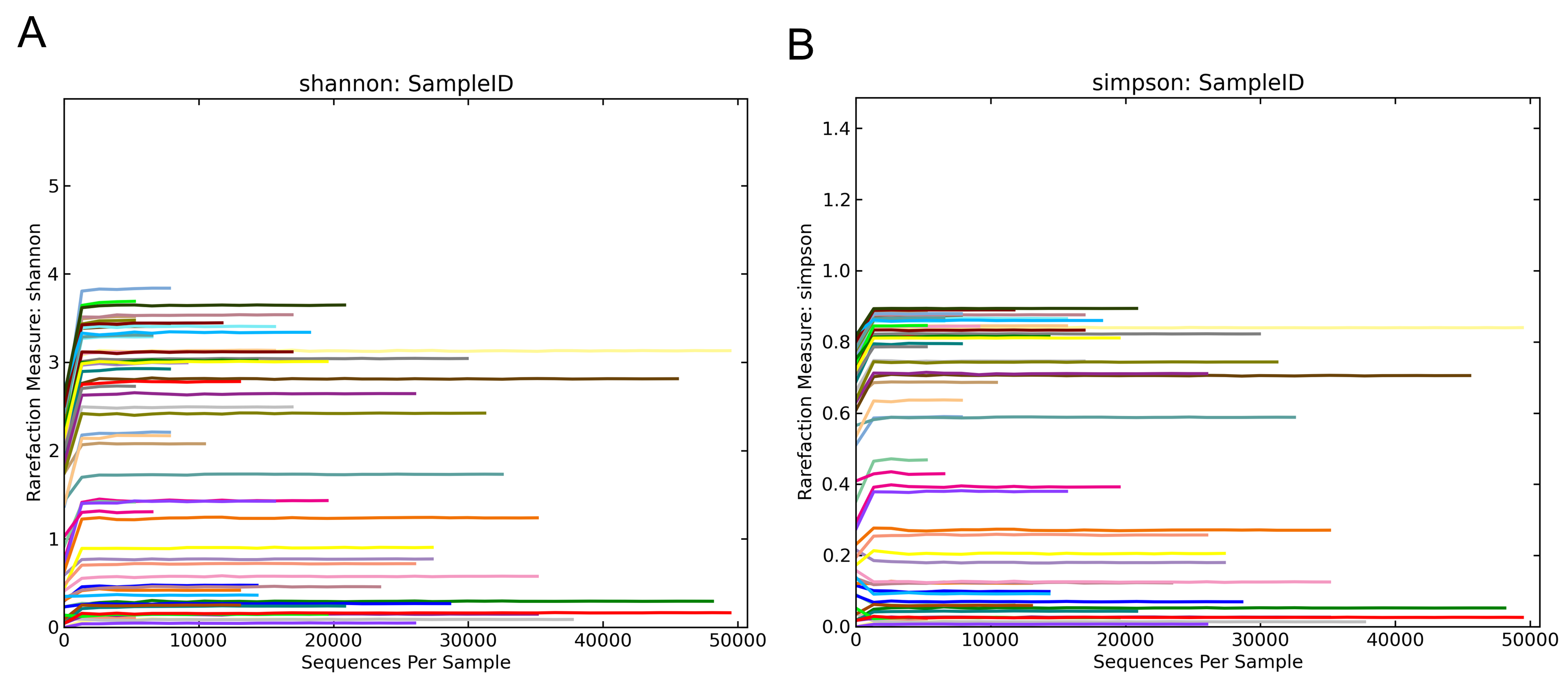

Supplement: Figure S2 — Rarefaction plots based on: (A) Shannon diversity index (a quantitative measure of community richness). (B) Simpson diversity index (a qualitative and quantitative measure of community richness). The alpha diversity was assessed at different sequencing depths. Each coloured-curve represents one of the 62 samples. [file peerj-07-7488-s002.png]

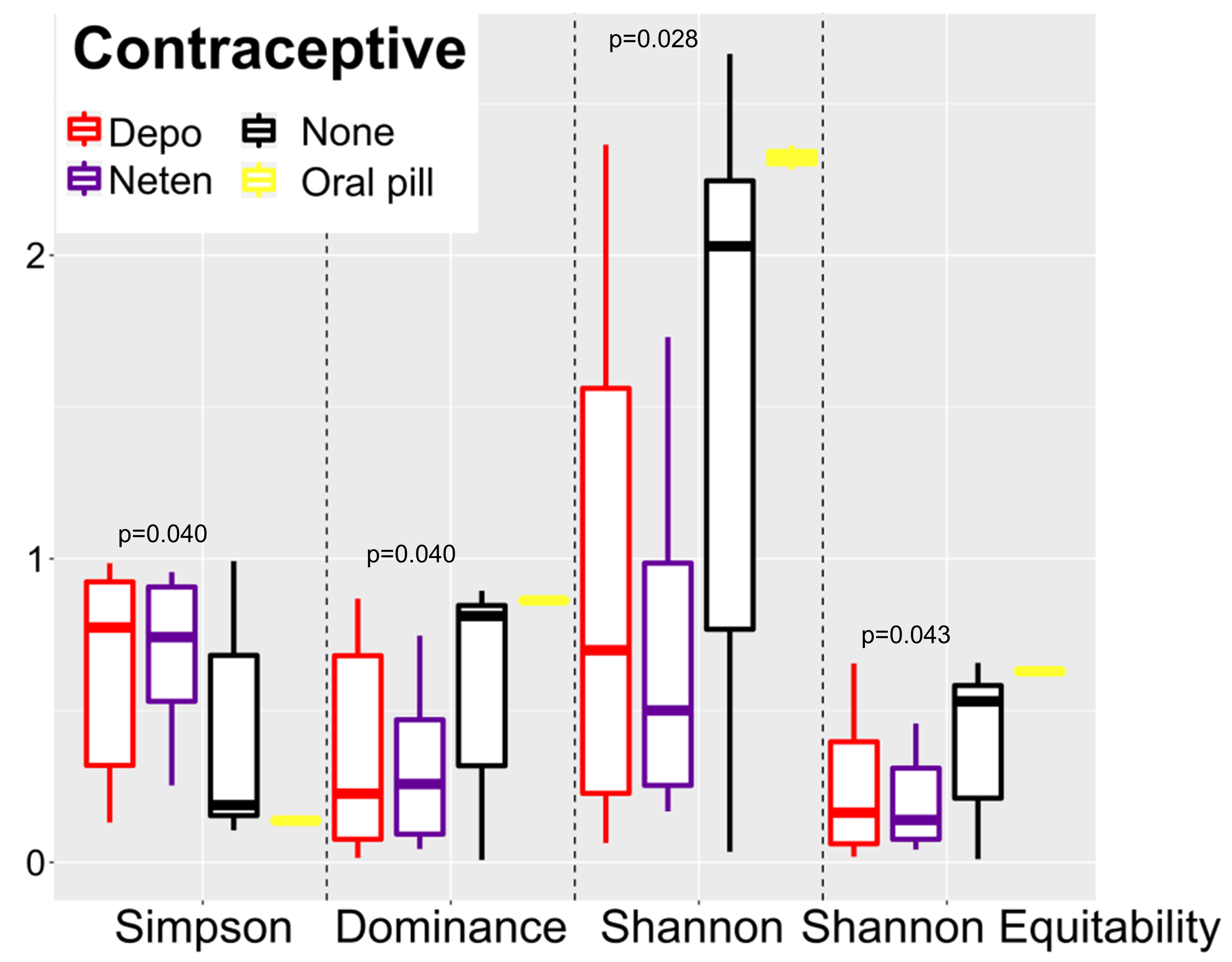

Supplement: Figure S3 — Each box plot is colour-coded according to the type of hormonal contraception. In each plot, the box ranges from the first to the third quartile, with the median represented by the line that divides the box into two. The whiskers extend to adjacent values within the lower and upper quartiles. Outliers are represented by the dots. Hormonal contraceptive users are categorized according to the type of contraceptive: oral (oral pills) or injectable (Depo: Depo-Provera and Neten: norethisterone enanthate) contraceptives. Besides being displayed in this figure, women in the oral pills group were not included in the calculation of the alpha diversity due to the small sample size in this group (n = 2). [file peerj-07-7488-s003.png]
