# Supplementary material for: Factors associated with the composition and diversity of the cervical microbiota of reproductive-age Black South African women: a retrospective cross-sectional study
Source: PeerJ. 2019 Aug 15;7:e7488. doi: 10.7717/peerj.7488 (PMC6698374; doi:10.7717/peerj.7488)
Supplement: Table S1 [file peerj-07-7488-s004.docx]

| **Step** | **QIIME/UPARSE** | **Script/Command Name** | **Parameters** |
| --- | --- | --- | --- |
| **Demultiplexing and quality filtering** | QIIME | split_libraries.py | -s, --min_qual_score (25); -b, --barcode_type (variable_length); -l, --min_seq_length (200); L, --max_seq_length (400); -d, --record_qual_scores; -j, --added_demultiplex_field (run_prefix); w, --qual_score_window (50); --z, reverse_primers (truncate_only) |
| **Quality filtering using UPARSE** | UPARSE | usearch --fastq_filter | E, --fastq_maxee (0.5); L, --fastq_minlen 270 |
| **Dereplicating sequences** | UPARSE | usearch --derep_fulllength | - |
| **Abundance sorting and discarding singletons** | UPARSE | usearch --sortbysize | --minsize (2) |
| **UPARSE-OTU method for OTU clustering with the greedy clustering algorithm and representative sequence picking with abundance algorithm** | UPARSE | usearch --cluster_otus | --otu_radius_pct (3.0) |
| **Reference chimera checking and removal using uchime** | UPARSE | usearch --uchime_ref | --mindiv (0.8); mindiffs (3); --db Greengenes, gg13_8 |
| **Mapping original quality-filtered reads back to OTUs** | UPARSE | usearch --usearch_global | --id (0.97) |
| **Assigning taxonomy to OTUs using RDP Classifier** | QIIME | assign_taxonomy.py | -t Greengenes gg13_8 |
| **Sequence alignment** | QIIME | align_seqs.py | -e, --min_length (75); -p, --minimum_percent_id (0.75) |
| **Filtering sequence alignment** | QIIME | filter_alignment.py | - g, --allowed_gap_frac (0.999999) |
| **Inferring phylogeny** | QIIME | make_phylogeny.py | - |
| **Generating a dense/sparse representation of an OTU table (observation matrix)** | QIIME | make_otu_table.py | -e, --exclude_otus_fp |
| **Diversity analyses** | QIIME | core_diversity_analyses.py | -e, --sampling_depth (5000); -c, --categories; -p, --parameter_fp (metrics: chao1, observed_species, Shannon, simpson, PD_whole_tree, unweighted_unifrac,weighted_unifrac,bray_curtis; single rarefaction depth: 5000) |
