# Supplementary material for: Factors associated with the composition and diversity of the cervical microbiota of reproductive-age Black South African women: a retrospective cross-sectional study
Source: PeerJ. 2019 Aug 15;7:e7488. doi: 10.7717/peerj.7488 (PMC6698374; doi:10.7717/peerj.7488)
Supplement: Table S2 — Abbreviation: OTU operational taxonomic unit. [file peerj-07-7488-s005.docx]

| **Bacterial OTU** | **Prevalence (% (n/N))** | **Mean relative abundance (%)** |
| --- | --- | --- |
| ***L. iners*** | 100.0 (62/62) | 37.6 |
| ***Gardnerella* sp.** | 87.1 (54/62) | 9.8 |
| ***Shuttleworthia* sp.** | 46.8 (29/62) | 6.3 |
| ***Sneathia* sp.** | 80.6 (50/62) | 5.3 |
